# Tandem attenuators control expression of the *Salmonella mgtCBR* virulence operon

**DOI:** 10.1111/j.1365-2958.2012.08188.x

**Published:** 2012-10

**Authors:** Eun-Jin Lee, Eduardo A Groisman

**Affiliations:** 1 Howard Hughes Medical Institute, Yale School of Medicine, Department of Microbial Pathogenesis, Boyer Center for Molecular Medicine295 Congress Avenue, New Haven, CT 06536-0812, USA; 2 Yale Microbial Diversity InstitutePO Box 27389, West Haven, CT 06516, USA

## Abstract

The *mgtCBR* operon from *Salmonella enterica* serovar Typhimurium specifies the virulence protein MgtC, the Mg^2+^ transporter MgtB and the regulatory peptide MgtR. The *mgtCBR* transcript includes a long leader region harbouring two short open reading frames (ORFs). Translation of these ORFs is anticipated to impact the formation of particular stem-loop structures and control transcription of the coding region by an attenuation-like mechanism. We previously reported that ORF *mgtM* enables *Salmonella* to promote transcription of the *mgtC* and *mgtB* coding regions when experiencing a rise in cytoplasmic ATP levels. We now show that the proline codon-rich ORF *mgtP* mediates an increase in transcription of the *mgtC* and *mgtB* coding regions under conditions predicted to decrease the levels of proline-charged tRNA^Pro^. The high ATP and low proline signals act independently in an additive form. Replacing conserved *mgtP* proline codons with codons specifying other amino acids abolished the response to proline limitation but had no effect on the response to ATP. Substitution of conserved adenine nucleotides in *mgtM* abolished the response to ATP but had no effect in the response to proline limitation. This provides a singular example of a leader mRNA with tandem attenuators responding to different signals.

## Introduction

Transcription attenuation is a bacterial regulatory mechanism that entails the formation of either of two alternative based-paired RNA structures in the leader region of a transcript: one promoting transcription termination and one advancing transcription elongation into the coding region. Which RNA structure forms is determined by growth conditions that favour (or hinder) the normal coupling of transcription of the leader region with translation of a short open reading frame (ORF) located within the leader (Landick *et al*., 1996; Henkin and Yanofsky, 2002; Merino and Yanofsky, 2005; Grundy and Henkin, 2006; Naville and Gautheret, 2009). Normally, a single attenuator responding to a specific signal controls expression of genes involved in nutrient biosynthesis. Here we describe an unusual leader mRNA with tandem attenuators, each responding to a different signal, which dictate genetic control of an operon involved in virulence and metal homeostasis.

The *mgtCBR* operon from *Salmonella enterica* serovar Typhimurium specifies the virulence protein MgtC, which is required for survival inside macrophages (Blanc-Potard and Groisman, 1997); the P-type ATPase MgtB, which transports Mg^2+^ from the periplasm to the cytoplasm (Snavely *et al*., 1991); and the peptide MgtR, which promotes the FtsH-mediated proteolysis of MgtC (Alix and Blanc-Potard, 2008). MgtR also promotes degradation of the Mg^2+^ transporter MgtA (Choi *et al*., 2012), which is specified somewhere else in the genome (Maguire, 2006). Unlike MgtC, MgtB and MgtR are not necessary to cause a lethal infection in BALB/c mice or to survive within the macrophage-like cell line J774.1 (Blanc-Potard and Groisman, 1997; Alix and Blanc-Potard, 2008).

Expression of the *mgtCBR* operon is regulated at multiple levels. Transcription initiation requires the PhoP/PhoQ two-component system (Soncini *et al*., 1996), which is activated when bacteria experience low Mg^2+^ (Garcia Vescovi *et al*., 1996), acidic pH (Prost *et al*., 2007) and/or certain antimicrobial peptides (Bader *et al*., 2005). Interestingly, PhoP also promotes transcription of AmgR, an anti-sense RNA for the *mgtC* portion of the polycistronic *mgtCBR* message (Lee and Groisman, 2010). Transcription elongation into the coding region is controlled by the 296 nt long mRNA leader, which responds to low cytosolic Mg^2+^ (Cromie *et al*., 2006; Spinelli *et al*., 2008) and to an increase in cytosolic ATP levels (Lee and Groisman, 2012) by advancing transcription of the *mgtC* and *mgtB* coding regions.

The response to cytosolic ATP requires a stretch of adenine nucleotides in a region of the mRNA leader located within a short ORF designated *mgtM* (Lee and Groisman, 2012). The deduced amino acid sequence of *mgtM* is not conserved in the mRNA leader of *mgtC* homologues. By contrast, the presence of adenine nucleotides and their location relative to regions with the potential to adopt particular stem-loop structures are conserved in the *mgtCBR* leader. The mRNA levels corresponding to the *mgtC* and *mgtB* coding regions increase dramatically when *Salmonella* is inside macrophages. This increase, and *Salmonella*’s ability to cause a lethal infection in mice, is dependent, in part, on the conserved adenine nucleotides in the *mgtCBR* leader mediating the response to ATP (Lee and Groisman, 2012).

The *Salmonella mgtA* gene specifies a protein that is 50% identical to MgtB (Maguire, 2006). Like *mgtB*, the *mgtA* gene is transcribed from a PhoP-dependent promoter (Garcia Vescovi *et al*., 1996) and harbours a Mg^2+^-responding mRNA leader (Cromie *et al*., 2006; Spinelli *et al*., 2008). Unlike *mgtB,* the *mgtA* coding region is not induced inside macrophages (Lee and Groisman, 2012), and this could be due to the absence of conserved adenine nucleotides in the *mgtA* mRNA leader (Lee and Groisman, 2012).

We now describe the identification of a short ORF rich in proline codons in the *mgtCBR* mRNA leader that enables *Salmonella* to stimulate transcription of the *mgtC* and *mgtB* coding regions under conditions that decrease the levels of proline-charged tRNA^Pro^. This is reminiscent of the expression behaviour of the *mgtA* coding region, which is also regulated by changes in proline-charged tRNA^Pro^ sensed by an unrelated proline codon-rich ORF in the *mgtA* leader region (Park *et al*., 2010). The *mgtCBR* leader provides a singular example of a leader mRNA with tandem attenuators responding to distinct signals: ATP and proline.

## Results

### The *mgtCBR* leader *mRNA* harbours a translated proline codon-rich short ORF

**Figure 1:**
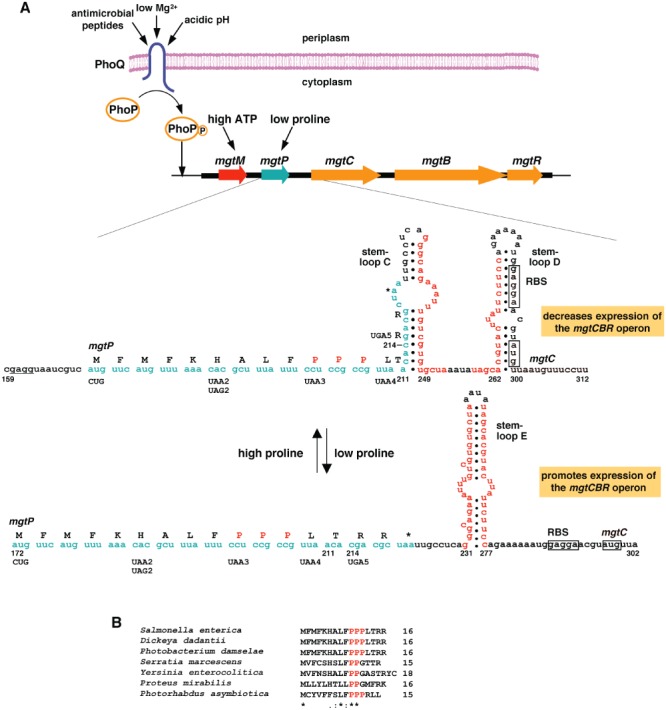
Regulation of the *Salmonella mgtCBR* virulence operon by the PhoP/PhoQ system and *mgtCBR* leader region. A. The sensor PhoQ responds to extracytoplasmic low Mg^2+^, acidic pH and antimicrobial peptides by promoting phosphorylation of the PhoP protein, which binds to the *mgtCBR* promoter resulting in transcription initiation. Transcription elongation into the coding region is controlled by the leader region, which can adopt alternative secondary structures depending on the coupling/uncoupling of transcription of the *mgtCBR* leader and translation of two short ORFs designated *mgtM* and *mgtP*. Which secondary structures form is determined by the cytoplasmic levels of ATP and proline. The alternative secondary structures potentially adopted by the segment of the *mgtCBR* leader that includes the *mgtP* ORF up to the second *mgtC* codon (i.e. stem-loops C and D versus stem-loop E) are shown with the predicted RBS for *mgtP* underlined. The *mgtP* sequences are indicated in cyan. The predicted RBS and *mgtC* start codon are boxed. Positions and sequences of stop codon mutations or nucleotide substitutions in the strains used in the experiments presented in Fig. 2 are indicated below the *mgtP* sequence. B. Alignment of the deduced amino acid sequences of *mgtP* in the *mgtCBR* leader regions from *Salmonella enterica*, *Dikeya dadantii*, *Photobacterium damselae*, *Serratia marcescens*, *Yersinia enterocolitica*, *Proteus mirabilis*, and *Photorhadus asymbiotica*. Sequences in red correspond to Pro codons. Asterisks correspond to positions conserved in all listed species.

We analysed the *mgtCBR* leader region seeking sequence elements that may suggest it can sense additional signals. We found a 17-codon long ORF that includes three consecutive proline codons and is preceded by a sequence resembling a Shine–Dalgarno sequence 9 nt from the putative start codon (Fig. 1A). This ORF, designated *mgtP*, is located in a region of the leader RNA that has the potential to adopt two alternative secondary structures: stem-loops C and D versus stem-loop E (Fig. 1A). Because the last four *mgtP* codons are part of the left arm of stem-loop C, translation of the complete *mgtP* is predicted to hinder formation of stem-loop C and to favour formation of stem-loop E. The presence of a proline codon-rich short ORF and its location relative to the stem-loop structures just discussed are conserved in the predicted leader mRNA regions of *mgtC* homologues (Fig. S1).

We verified the formation of stem-loops C and D using in-line probing with a labelled RNA corresponding to nucleotides 196–385 [relative to the *mgtC* transcription start site ([Bibr b22][Bibr b22]; Zwir *et al*., 2012)]. The spontaneous RNA cleavage at regions not predicted to be part of stems differed in RNAs incubated in the presence of 1 mM versus 5 or 20 mM Mg^2+^. For instance, regions 1 and 4 were more accessible to cleavage at low than at high Mg^2+^ and the converse was true for regions 2, 3, 5 and 6 (Fig. S2). These data indicate that Mg^2+^ can modify the structure of this portion of the *mgtCBR* leader, which provides support for the genetic experiments suggesting that the *mgtCBR* leader functions as a Mg^2+^-sensing RNA (Cromie *et al*., 2006; Spinelli *et al*., 2008).

To examine whether *mgtP* is translated *in vivo*, we utilized our previously reported approach (Park *et al*., 2010) to determine the β-galactosidase activity produced by wild-type *Salmonella* harbouring a plasmid that expressed an *mgtP–lacZ* translational fusion and included the predicted Shine–Dalgarno sequence for *mgtP*. As a control, we used an isogenic derivative in which the truncated *lacZ* gene was placed after the *mgtP* stop codon. The former strain produced high levels of β-galactosidase whereas no activity was observed for the latter (Fig. S3). Taken together with our previous findings (Lee and Groisman, 2012), these results indicate that the *mgtCBR* leader mRNA includes two short ORFs, the translation of which is predicted to impinge the formation of particular stem-loop structures.

### *mgtP* is part of a transcription attenuator

The possibility of the *mgtCBR* leader mRNA adopting alternative stem-loop structures (stem-loops C and D versus stem-loop E) in the region overlapping and adjacent to *mgtP* (Fig. 1A) suggested that *mgtP* might control transcription elongation into the *mgtCBR* coding region by a transcription attenuation mechanism similar to those governing expression of biosynthetic operons in enteric bacteria (Merino and Yanofsky, 2005). If this is the case, uncoupling transcription of the *mgtCBR* leader region and translation of *mgtP* might affect expression of the associated coding regions. To test this idea, we determined the β-galactosidase activity in a set of isogenic strains harbouring a *lac* transcriptional fusion in the chromosomal *mgtC* coding region and a wild-type or mutant *mgtP*.

**Figure 2:**
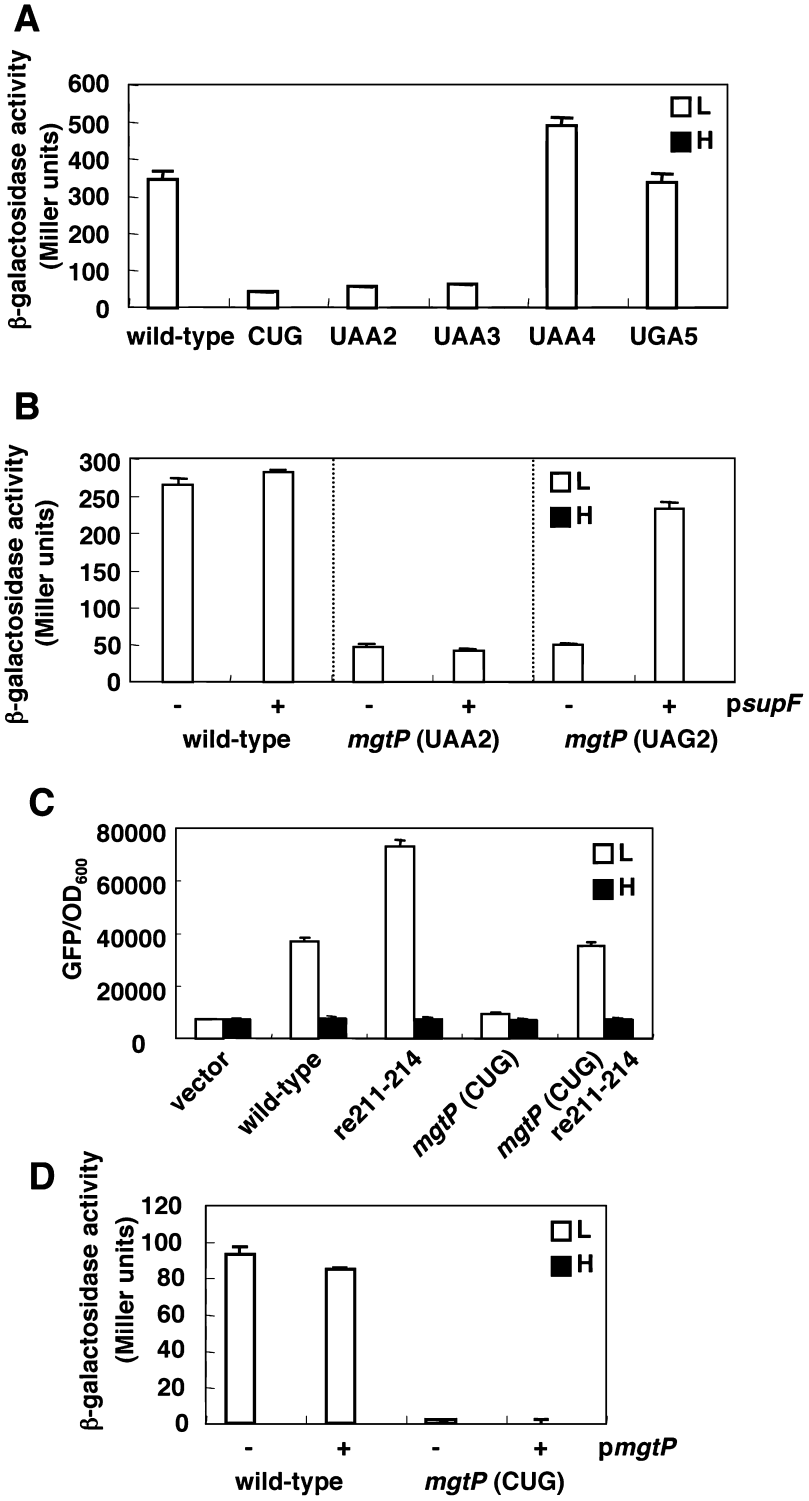
*mgtCBR* leader controls expression of the *mgtCBR* coding region by a transcription attenuation-like mechanism. A. β-Galactosidase activity (Miller units) produced by a *Salmonella* strain with a chromosomal *mgtC–lac* fusion (EG9527) and isogenic derivatives with mutation of the start codon (EG18799) or with stop codons at different positions (EG18801, EG19251, EG19272 and EG19253) in *mgtP*. Bacteria were grown in N-minimal media containing low (L; 10 µM) or high (H; 10 mM) Mg^2+^ for 4 h. Shown are the mean and SD from at least three independent experiments. B. β-Galactosidase activity (Miller units) produced by a *Salmonella* strain with a chromosomal *mgtC-lac* fusion (EG9527) harbouring either plasmid p*supF* or the empty vector pUH21-2*lacI*^q^ or by isogenic derivatives with an amber stop codon (EG19840) or ochre stop codon (EG18801) at position 187–189 (i.e. *mgtP*’s 6th codon). Bacteria were grown as described above except in the presence of ampicillin (50 µg ml^−1^) and IPTG (0.2 mM). C. Fluorescence produced by wild-type *Salmonella* (14028s) harbouring a plasmid encoding a translational fusion to *gfp* and the wild-type *mgtC* leader, or derivatives with mutations that hinder stem-loop C formation (re211-214) and/or with mutation of the *mgtP* start codon. Bacteria were grown as described above except in the presence of ampicillin (50 µg ml^−1^). Shown are the mean and SD from at least three independent experiments. D. β-Galactosidase activity (Miller units) produced by a *Salmonella* strain with a chromosomal *mgtC-lac* fusion (EG9527) or an isogenic strain with mutation of the *mgtP* start codon (EG18799) harbouring either the plasmid vector or plasmid p*mgtP*. Bacteria were grown as described above except in the presence of ampicillin (50 µg ml^−1^) and IPTG (0.1 mM). Shown in (B) and (D) are the mean and SD from two independent experiments. For parts A, B and D, the activity was lower than the resolution of the figure following growth in high (H; 10 mM) Mg^2+^.

A strain in which the *mgtP* start codon was changed from AUG to CUG produced seven times less β-galactosidase than the isogenic *mgtP^+^* strain when grown in low Mg^2+^ to induce the PhoP/PhoQ system (Fig. 2A). Strains with stop codon mutations at the 6th or 10th positions of *mgtP* expressed similar low levels of β-galactosidase (Fig. 2A). We ascribe the low *mgtC–lac* expression of these mutants to a defect in *mgtP* translation as opposed to a structural change that ‘locked’ the mRNA leader in a low expression conformation. This is because a plasmid expressing the amber suppressor *supF* restored wild-type levels of *mgtC–lac* transcription to an *mgtP* mutant with an amber stop codon at the 6th position but not to one harbouring an ochre stop codon at that position (Fig. 2B). As expected, the *supF*-expressing plasmid had no effect on *mgtC–lac* transcription in a strain harbouring the wild-type *mgtC* leader (Fig. 2B).

Derivatives with stop codon mutations at the 13th or 15th positions retained the expression behaviour of the strain with the wild-type leader (Fig. 2A). Given the space that a translating ribosome occupies on a transcript [12–15 nucleotides from the P site (Steitz, 1969)], *mgtP* translation beyond the 12th codon is anticipated to favour formation of stem-loop E. By contrast, when *mgtP* translation stops before the ribosome reaches the 13th *mgtP* codon, stem-loops C and D would form (Fig. 1), which reduces expression of the *mgtCBR* coding region.

To further address the role of *mgtP* translation in *mgtC* expression, we measured fluorescence in wild-type *Salmonella* harbouring a plasmid in which the PhoP-dependent *mgtC* promoter, full-length *mgtCBR* leader and first two codon of *mgtC* gene were fused in frame to the third codon of the *gfp* gene. Fluorescence was fivefold higher following growth in low versus high Mg^2+^ (Fig. 2C). This is likely due to the PhoP-dependent promoter and Mg^2+^-responding mRNA leader because the expression levels were similarly low in an isogenic strain carrying the plasmid vector (Fig. 2C). A strain with a plasmid derivative in which the *mgtP* start codon AUG was substituted for CUG displayed decreased fluorescence (Fig. 2C), in agreement with the phenotype of a strain with the equivalent mutation in the chromosomal copy of *mgtP* (Fig. 2A). By contrast, a strain with a plasmid derivative substituted in nucleotides 211–214 of the *mgtCBR* leader exhibited higher fluorescence than the strain with the plasmid harbouring the wild-type *mgtCBR* leader (Fig. 2C). This mutation is expected to disrupt formation of stem-loop C, favour formation of stem-loop E, and release the ribosome binding site (RBS) and *mgtC* start codon sequestered in stem-loop D (Fig. 1). The substitution of the 211–214 region could overcome (partially) the decrease in expression resulting from mutation of the *mgtP* start codon (Fig. 2C).

Finally, a plasmid carrying the *mgtP* ORF failed to restore normal expression to a strain with a chromsomal *mgtC–lac* transcriptional fusion and a mutation of the *mgtP* start codon, behaving like the vector control (Fig. 2D); and it had no effect on an isogenic strain with a wild-type *mgtC* leader (Fig. 2D). This argues against the notion of *mgtP* exerting its regulatory effect by specifying a *trans-*acting peptide. Cumulatively, the results presented here indicate that *mgtP* acts as a *cis*-regulatory element*,* regulating the associated *mgtCBR* coding region by being part of a transcription attenuator.

### Intracellular proline controls expression of the *mgtC* and *mgtB* coding regions dependent on conserved *mgtP* proline codons

We postulated that *mgtP* might confer regulation by intracellular proline levels because it includes three consecutive Pro codons, which is a disproportionately high frequency for a 17-codon long ORF (McCaldon and Argos, 1988), and also because the presence of consecutive Pro codons near the base of stem-loop C is conserved in the *mgtCBR* leader of other species (Figs 1 and S1). Furthermore, an unrelated short ORF harbouring four proline codons in the *mgtA* leader mRNA mediates expression of the *mgtA* coding region in response to changes in the levels of cytosolic proline (Park *et al*., 2010). Thus, conditions that decrease the levels of cytosolic proline available to charge tRNA^Pro^ may result in the ribosome stalling at the *mgtP* Pro codons thereby advancing a structure that alters transcription of the *mgtCBR* coding region.

**Figure 3:**
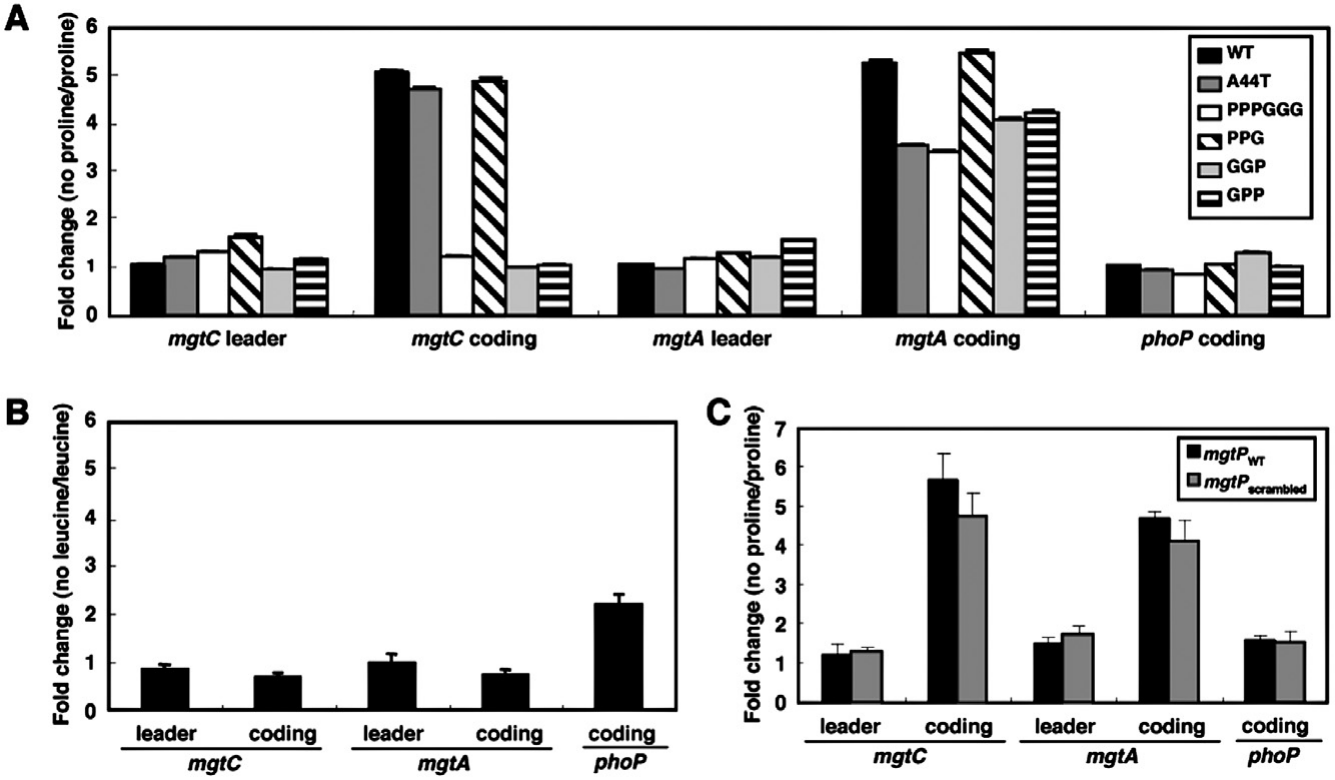
Proline limitation promotes transcription of the *mgtCBR* coding region in a manner dependent on conserved Pro codons in *mgtP*. A. Fold change in the mRNA levels of the leader regions of the *mgtC* and *mgtA* transcripts and the coding regions of the *mgtC*, *mgtA*, and *phoP* genes produced by a proline auxotroph harbouring either the wild-type *mgtCBR* leader (EL302), or derivatives where the A nucleotides at position 44–46 were substituted by Ts (A44T; EL339), or where the three *mgtP* Pro codons (PPPGGG; EL303), the Pro codons at the 12th (PPG; EL347), 10th and 11th (GGP; EL348) or 10th (GPP; EL349) positions were substituted by Gly codons. Bacteria were grown in N-minimal media with 500 µM Mg^2+^ in the presence of 1 mM proline for 1 h, and then grown for 45 min in media containing or lacking proline. Expression levels of target genes were normalized to that of 16S ribosomal RNA *rrs* gene. Fold change was calculated by dividing the mRNA levels of cells grown in the absence of proline by that of cells grown in the presence of proline. Shown are the mean and SD from three independent experiments. B. Fold change in the mRNA levels of the leader regions of *mgtC* and *mgtA* transcripts and the coding regions of the *mgtC*, *mgtA*, and *phoP* genes produced by a leucine auxotroph (EL337) under leucine limitation conditions analogous to that described above for proline limitation. Shown are the mean and SD from two independent experiments. C. Fold change in the mRNA levels of the genes listed above produced by a proline auxotroph with a wild-type *mgtCBR* leader (EG19886) sequence or with the *mgtP* sequence scrambled (EL379) following growth as described in (A). Shown are the mean and SD from two independent experiments.

We determined that the mRNA corresponding to the *mgtC* coding region was present at fivefold higher levels in a proline auxotroph grown in the absence of proline for 45 min than when it was grown in its presence (Fig. 3A). Proline limitation specifically induced *mgtC* expression in a manner dependent on the *mgtP* Pro codons because: first, it failed to promote an increase in the mRNA levels corresponding to the *phoP* coding region or the *mgtCBR* and *mgtA* leader regions (Fig. 3A). However, it did induce the *mgtA* coding region (Fig. 3A), which was used as a positive control (Park *et al*., 2010). Second, *mgtC* mRNA levels did not increase in a leucine auxotroph subjected to leucine limitation (Fig. 3B) despite the fact that *mgtP* includes two leucine codons (Fig. 1). And third, substitution of the three *mgtP* Pro codons by Gly codons prevented *mgtC* induction in response to proline limitation (Fig. 3A). As expected, the latter *mgtP* mutant retained wild-type expression of the *phoP* coding region and the *mgtCBR* and *mgtA* leader regions and still induced the *mgtA* coding region in response to proline limitation (Fig. 3A).

Derivatives of the *mgtCBR* leader with substitutions of the *mgtP* Pro codons at the 10th position or the 10th and 11th positions failed to promote *mgtC* expression upon proline limitation (Fig. 3A). By contrast, a mutant with a substitution of the Pro codon at the 12th position exhibited a wild-type behaviour (Fig. 3A). This was expected given that *mgtP* harbours only two Pro codons in certain bacterial species (corresponding to the 10th and 11th positions; Fig. 1B). Furthermore, a strain in which the *mgtP* sequence was completely scrambled (except for the Pro codons at the 10th and 11th positions and the sequence required for formation of stem-loop C) still responded to proline limitation by enhancing the mRNA levels of the *mgtC* coding region (Fig. 3C). In sum, these experiments indicate that the *mgtP* Pro codons at the 10th and 11th positions are necessary, and possibly sufficient, for low cytosolic proline to induce transcription of the *mgtC* and *mgtB* coding regions.

### Hyperosmotic stress promotes expression of the *mgtC* and *mgtB* coding regions

**Figure 4:**
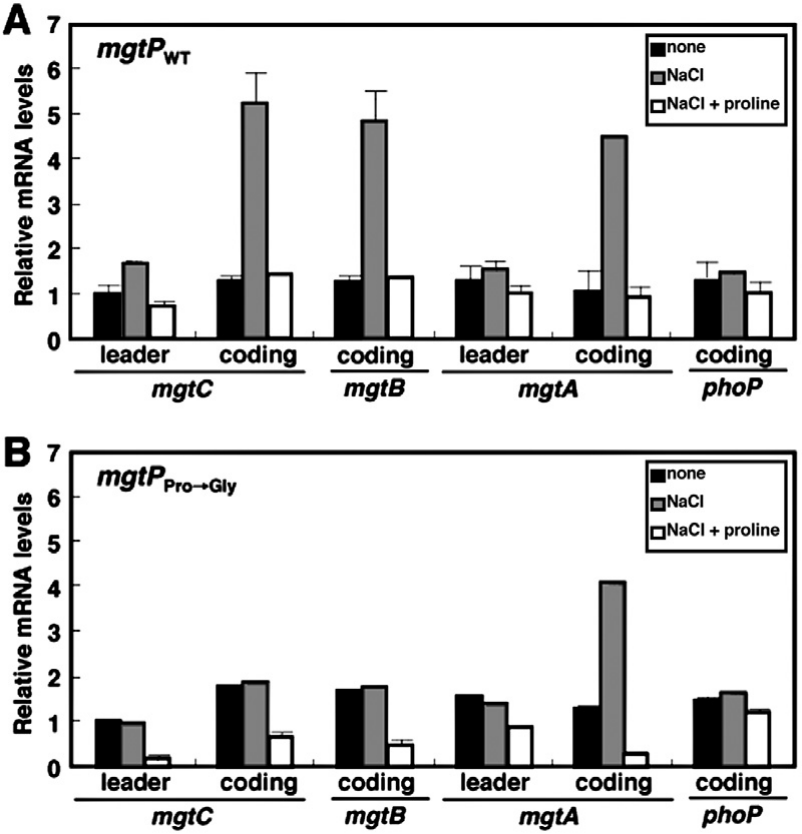
Hyperosmotic stress promotes transcription of the *mgtCBR* coding region. mRNA levels of the leader regions of the *mgtC* and *mgtA* transcripts and the coding regions of the *mgtC*, *mgtA*, and *phoP* genes in strains with the wild-type *mgtCBR* leader (EL296) or a derivative with the three *mgtP* Pro codons substituted by Gly codons (EL304). The RNA values were normalized relative to those corresponding to the *rrs* gene. Bacteria were grown for 1 h in modified N-minimal medium without casamino acids and containing 500 µM Mg^2+^, or in media that also had 0.3 M NaCl, or 0.3 M NaCl and 1 mM proline. Shown are the mean and SD from three independent experiments.

In addition to being a component of peptides and proteins, proline can function as an osmoprotectant (Csonka and Leisinger, 2007). This raised the possibility of hyperosmotic stress promoting transcription of the *mgtCBR* coding region by virtue of decreasing the amount of cytosolic proline available to charge tRNA^Pro^. Indeed, when wild-type *Salmonella* experienced 0.3 M NaCl, the mRNA corresponding to the *mgtC* and *mgtB* coding regions increased fivefold (Fig. 4). This is similar to the induction of the *mgtA* coding region (Fig. 4), as we previously reported (Park *et al*., 2010). The increase in the mRNA levels of the *mgtC* and *mgtB* coding regions (but not that corresponding to the *mgtA* coding region) is dependent on the *mgtP* Pro codons (Fig. 4). By contrast, hyperosmotic stress had no effect on the transcript levels of the *phoP* coding region or the *mgtCBR* and *mgtA* leader regions (Fig. 4). As expected, the induction resulting from hyperosmotic stress was eliminated when proline was present in the media (Fig. 4). Thus, the Mg^2+^ transporter-specifying *mgtA* and *mgtB* genes rely on different Pro codon-rich short ORFs in their mRNA leaders to induce their respective coding regions under hyperosmotic stress.

### The proline and ATP signals act on the *mgtCBR* leader independently and additively

**Figure 5:**
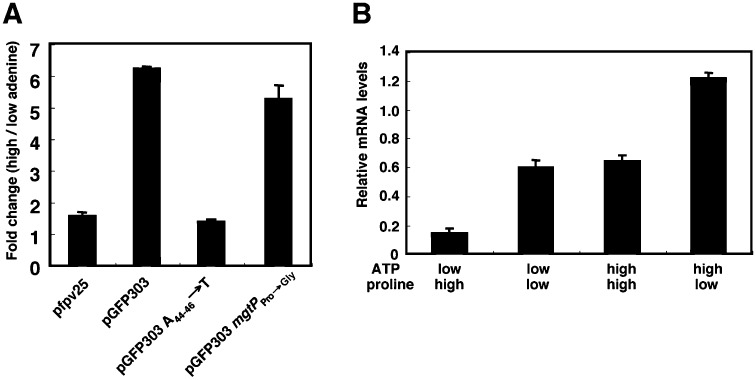
High ATP and low proline promote transcription of the *mgtCBR* coding region in an independent and additive manner. A. Fluorescence produced by an adenine auxotroph (EG9652) harbouring plasmid pGFP303 with the PhoP-dependent *mgtCBR* promoter and wild-type *mgtCBR* leader fused to a promoterless *gfp* gene, the plasmid vector pfpv25, or pGFP303 derivatives with conserved A nucleotides at position 44–46 substituted by Ts (pGFP303 A_44–46_→T) or with the three *mgtP* Pro codons substituted by Gly codons (pGFP303 *mgtP*_Pro→Gly_). Bacteria were grown in N-minimal media with 10 µM Mg^2+^ in the presence of either 25 µM or 250 µM adenine. Fluorescence was monitored following growth for 6.5 h with shaking at 37°C under microaerophilic conditions in a Victor^3^ plate reader. Data correspond to a representative of four independent experiments. B. mRNA levels of the coding regions of the *mgtC* gene produced by a proline and adenine auxotroph (EL333) grown under different combinations of high and low levels proline and adenine. The RNA values were normalized relative to those corresponding to the *rrs* gene. Bacteria were grown in N-minimal media with 500 µM Mg^2+^ in the presence of 1 mM proline and 25 µM adenine for 1 h, and then grown for 1 h in media either containing or lacking proline, 25 µM or 250 µM adenine.

As discussed above, upstream of *mgtP* there is an additional short ORF, designated *mgtM* and including conserved adenine nucleotides, which enables an increase in intracellular ATP levels to promote transcription of the *mgtC* and *mgtB* coding regions (Lee and Groisman, 2012). We investigated whether the ability to respond to proline is independent from that mediating the response to ATP by exploring the response to these two signals in strains with mutations in the conserved regions of *mgtM* and *mgtP*. A chromosomal mutant with the adenine nucleotides at position 44–46 of the *mgtCBR* leader substituted for thymine nucleotides exhibited normal derepression of the *mgtC* coding region provoked by proline limitation (Fig. 3A) even though it no longer responded to an increase in ATP levels (Fig. 5A) (Lee and Groisman, 2012). Likewise, a chromosomal mutant in which the *mgtP* Pro codons were substituted by Gly codons displayed wild-type capacity to respond to changes in the adenine concentration in the media (Fig. 5A) despite lacking the ability to respond to proline (Fig. 3A).

The effect of the low proline and high ATP signals is additive because the mRNA levels corresponding to the *mgtC* coding region were nearly two times higher in wild-type *Salmonella* experiencing both conditions than those subjected to either proline limitation or high levels of ATP alone (Fig. 5B). This provides a singular example of a leader mRNA that harbours tandem attenuators responding to different signals.

## Discussion

We established that the *mgtCBR* leader mRNA is a complex sensing device that can respond to a variety of signals by altering the expression of a virulence protein and a Mg^2+^ transporter. On the one hand, it relies on the coupling of transcription of the *mgtCBR* leader mRNA and translation of two short ORFs located within the leader to alter the mRNA levels of the associated coding regions in response to the cytoplasmic levels of ATP and proline. On the other hand, the *mgtCBR* leader mRNA can respond to cytoplasmic Mg^2+^ (Cromie *et al*., 2006; Spinelli *et al*., 2008), perhaps acting as a Mg^2+^-sensing riboswitch like those preceding the coding regions of the Mg^2+^ transporter genes *mgtA* in *Salmonella* (Cromie *et al*., 2006) and *mgtE* in *Bacillus subtilis* (Dann *et al*., 2007).

### Tandem attenuators in the *mgtCBR* leader mediate responses to two different signals

Classical attenuators regulate the expression of products that alter the levels of a metabolite affecting the coupling of transcription of the leader region and translation of an ORF located within the leader mRNA. For example, the transcript corresponding to the *trp* operon from *Escherichia coli*, which specifies enzymes responsible for tryptophan biosynthesis, includes a leader with a short ORF harbouring two adjacent Trp codons. This allows control of the genes specifying Trp biosynthetic enzymes by the levels of charged tRNA^Trp^ because when cytoplasmic tryptophan levels are low, ribosome stalling at the ORF Trp codons enables the leader RNA to adopt a conformation that favours transcription elongation into the *trp* operon coding region (Yanofsky, 2004). Likewise, the mRNA for the *E. coli pyrBI* operon, which encodes enzymes participating in the *de novo* synthesis of pyrimidine nucleotides, is preceded by a leader that includes a short ORF and an overlapping uridine-rich stretch. This allows intracellular UTP levels to alter the normal coupling of transcription and translation, and thus regulate expression of the pyrimidine biosynthetic genes (Turnbough and Switzer, 2008).

The *mgtCBR* leader mRNA is unusual both in harbouring two attenuators and in the nature of the signals that control these attenuators. We previously demonstrated that stop codon mutations in *mgtM* that resulted in translation of an ORF shorter than seven amino acids resulted in derepression of the *mgtC* coding region (Lee and Groisman, 2012). This was ascribed to formation of one stem-loop over the alternative stem-loop in the 5′ portion of the transcript (Lee and Groisman, 2012). It was also proposed that physiological conditions changing the intracellular ATP levels could affect the coupling/uncoupling between transcription of the *mgtCBR* leader and translation of *mgtM*. In other words, a ribosome-translating *mgtM* would be more likely to stay close to the RNA polymerase (RNAP) transcribing the *mgtCBR* leader when cytosolic ATP levels are low because RNAP may pause at the conserved adenine nucleotides hindering transcription elongation into the coding region. By contrast, *mgtCBR* transcription and *mgtM* translation could become uncoupled at high ATP levels, which would advance transcription into the coding region.

That mutation of the *mgtP* start codon mutant decreased expression of the *mgtC* coding region (Figs 1 and 2A) might be due to a genetic situation favouring formation of stem-loops C and D. This would also apply to the reduced expression displayed by strains with stop codon mutations at the 6th and 10th codons of *mgtP* (Fig. 2A). By contrast, stop codon mutations at 13th or 15th positions retained wild-type *mgtC*–*lac* expression. This is because the ribosome occupies 12–15 nt from P site (Steitz, 1969) and a ribosome translating beyond the 13th position would cover the left side of stem-loop C and favour formation of stem-loop E (Figs 1 and 2A). When bacteria experience physiological conditions that decrease the levels of cytosolic proline available to charge tRNA^Pro^, the ribosome would stall at the *mgtP* Pro codons thereby favouring formation of stem-loop E, which furthers transcription elongation into the *mgtCBR* coding region. Because formation of stem-loop D would sequester the RBS and start codon of *mgtC* (Fig. 1A), it is possible that *mgtP* translation affects translation of *mgtC* in addition to the transcriptional effects discussed above.

The vast majority of transcription attenuators described to date respond to a signal by affecting the formation of an intrinsic transcription terminator [i.e. a GC-rich stem-loop structure followed by a string of uridine nucleotides (Peters *et al*., 2011)]. However, the stem-loop structures identified in the *mgtCBR* leader mRNA (Lee and Groisman, 2012) (Fig. 1) do not resemble intrinsic transcription terminators. This is reminiscent of the *mgtA* leader, which also lacks an intrinsic transcription terminator. Given that the *mgtA* leader governs transcription elongation into the coding region by affecting access of the termination factor Rho (Hollands *et al*., 2012), this raises the possibility of a Rho-dependent terminator(s) controlling transcription elongation beyond the *mgtCBR* leader as well.

We determined that the ATP- and the proline-sensing attenuators present in the *mgtCBR* leader act independently and that the effect of the two inducing conditions is additive (Fig. 5). In other words, high ATP stimulates transcription of the *mgtC* and *mgtB* coding regions even when there is no proline limitation, and low proline stimulates *mgtC* and *mgtB* expression even if the ATP levels are not high. This bears interesting similarities and differences with the mRNA leader of the *B. clausii metE* gene, which harbours tandem riboswitches that monitor two different signals (i.e. *S*-adenosylmethionine and coenzyme B_12_) (Sudarsan *et al*., 2006). Like in the tandem attenuators in the *mgtCBR* leader, the tandem riboswitches in the *metE* leader work independently and the effect of the two signals is greater than that of each signal alone. Unlike the *mgtCBR* leader, the *metE* leader harbours two sites anticipated to function as intrinsic transcription terminators and the signals are sensed directly by the mRNA leader.

### Mg^2*+*^, hyperosmotic stress and ATP control transcription of the *mgtCBR* operon

Transcription initiation of the Mg^2+^ transporter loci *mgtA* and *mgtCBR* is controlled by the PhoP/PhoQ two-component system (Soncini *et al*., 1996), which is activated in low periplasmic Mg^2+^ (Garcia Vescovi *et al*., 1996). In addition, the *mgtA* and *mgtCBR* transcripts include long leader sequences that respond to low cytoplasmic Mg^2+^ by stimulating transcription of their respective coding regions (Cromie *et al*., 2006; Spinelli *et al*., 2008). This may allow *Salmonella* to differentially regulate expression of gene products that mediate cytoplasmic Mg^2+^ homeostasis (i.e. Mg^2+^ uptake systems) from those involved in extracytoplasmic Mg^2+^ homeostasis (i.e. proteins that modify Mg^2+^ binding sites in the bacterial cell surface).

We propose that hyperosmotic stress promotes expression of the *mgtB* and *mgtA* genes because both of them encode P-type ATPases (Snavely *et al*., 1991) that can transport Mg^2+^ even when *Salmonella* experiences a decrease in membrane potential (Csonka, 1989), a condition that compromises the activity of the constitutively expressed Mg^2+^ transporter CorA (Smith and Maguire, 1998). We previously reported that up-regulation of the *mgtA* coding region by hyperosmotic stress is mediated by a short ORF harbouring four Pro codons located in the *mgtA* leader region (Park *et al*., 2010). That the *mgtB* gene is also up-regulated by hyperosmotic stress (Fig. 4) and that it harbours an unrelated ORF with three consecutive Pro codons (Fig. 1) mediating the response to low proline (Fig. 3A) provides further support to the notion that *Salmonella*, and likely other enteric bacteria, utilize short ORFs rich in Pro codons to control expression of related Mg^2+^ transporters. Moreover, it argues against the proposal that the role of the Pro codon-rich ORF in the *mgtA* leader region is to mediate the response to low levels of cytoplasmic Mg^2+^ (Zhao *et al*., 2011).

The proline levels present at any given time in *Salmonella* result from the combined activities of proline biosynthetic enzymes and proline uptake systems (Csonka, 1989). The hyperosmotic stress induction of the *mgtC* and *mgtB* genes was detected in a proline prototroph with functional proline transporters during growth in the absence of casamino acids. Given that the total proline content does not change when *Salmonella* experiences hyperosmotic stress (Csonka, 1988), if proline is used for osmoprotection, then less proline would be available to charge tRNA^Pro^ with proline. This could then give rise to a situation where hyperosmotic stress results in ribosome stalling at the *mgtP* proline codons thereby resulting in transcription of the *mgtCB* coding region.

The similarities in expression behaviour discussed above for the *mgtA* and *mgtB* genes suggest that the corresponding Mg^2+^ transporters might be operating at the same time. However, this does not appear to be the case because: First, the PhoP-activated *mgtA* and *mgtCBR* promoters have different architectures (Zwir *et al*., 2012). Second, the *mgtC* and *mgtB* coding regions are induced under mild acidic pH conditions and inside macrophages whereas *mgtA*’s is not (Lee and Groisman, 2012). And third, the MgtR peptide specified in the *mgtCBR* operon has been shown to bind to the MgtA protein promoting its degradation (Choi *et al*., 2012). Given that the *mgtA* gene can be transcribed by the Rob protein independently of the PhoP/PhoQ system (Barchiesi *et al*., 2008), it appears that expression of the MgtA protein versus the MgtB protein is favour under different circumstances. This could reflect the 50% differences in amino acid identity between these transporters, which likely accounts for dissimilar substrate specificity (Maguire, 2006).

## Experimental procedures

### Bacterial strains, plasmids, oligodeoxynucleotides and growth conditions

Bacterial strains and plasmids used in this study are listed in Table 1. All *S. enterica* serovar Typhimurium strains are derived from the wild-type strain 14028s (Fields *et al*., 1986) and were constructed by phage P22-mediated transductions as described (Davis *et al*., 1980). All DNA oligonucletides are listed in Table S1. Bacteria were grown at 37°C in Luria–Bertani broth (LB), N-minimal media (Snavely *et al*., 1991) supplemented with 0.1% casamino acids, 38 mM glycerol and the indicated concentrations of MgCl_2_. To examine the effect of hyperosmotic stress on gene expression, we used a modified N-minimal medium containing 0.2% glucose instead of 38 mM glycerol. *Escherichia coli* DH5α was used as the host for preparation of plasmid DNA. Ampicillin was used at 50 µg ml^−1^, chloramphenicol was used at 20 µg ml^−1^, tetracycline at 10 µg ml^−1^ and fusaric acid at 12 µg ml^−1^.

**Table 1:** Bacterial strains and plasmids used in this study.

Strain or plasmid	Description	Reference or source
***S. enterica* serovar Typhimurium**		
14028s	Wild type	Fields *et al*. (1989)
TT206	LT2 *leu-1151*::Tn*10*	John R. Roth
EG9527	*mgtCB9232*::Mu*d*J	Blanc-Potard and Groisman (1997)
EG9652	*purB877*:: Tn*10*	Blanc-Potard and Groisman (1997)
EG18715	*mgtCB leader*::*tetRA*/pKD46	Lee and Groisman (2012)
EG18798	*mgtCBR leader*::*tetRA mgtC-lac*/pKD46	Lee and Groisman (2012)
EG18799	*mgtP* (CUG) *mgtC-lac*	This work
EG18801	*mgtP* (UAA2) *mgtC-lac*	This work
EG19251	*mgtP* (UAA3) *mgtC-lac*	This work
EG19253	*mgtP* (UGA5) *mgtC-lac*	This work
EG19272	*mgtP* (UAA4) *mgtC-lac*	This work
EG19840	*mgtP* (UAG2) *mgtC-lac*	This work
EG19886	*proB1657*::Tn*10*	Park *et al*. (2010)
EL296	*yicL*::Cm^R^	This work
EL302	*yicL*::Cm^R^, *proB1657*::Tn*10*	This work
EL303	*yicL*::Cm^R^*, mgtP* (Pro_10,11,12_ → Gly)*, proB1657*::Tn*10*	This work
EL304	*yicL*::Cm^R^*, mgtP* (Pro_10,11,12_ → Gly)	This work
EL333	*proB*::Cm^R^, *purB877*::Tn*10*	This work
EL337	*yicL*::Cm^R^, *leuB1151*::Tn*10*	This work
EL339	*yicL*::Cm^R^*, mgtM* (A_44-46_ → T), *proB1657*::Tn*10*	This work
EL347	*yicL*::Cm^R^*, mgtP* (Pro_12_ → Gly), *proB1657*::Tn*10*	This work
EL348	*yicL*::Cm^R^*, mgtP* (Pro_10,11_ → Gly), *proB1657*::Tn*10*	This work
EL349	*yicL*::Cm^R^, *mgtP* (Pro_10_ → Gly), *proB1657*::Tn*10*	This work
EL350	*proB*::Cm^R^	This work
EL379	*mgtP*_scrambled_, *proB1657*::Tn*10*	This work
**Plasmid**				
pACYC-′*lacZ*	rep_p15A_ Cm^R^*′lacZ*	Park *et al*. (2010)
pCP20	rep_pSC101_^ts^ Ap^R^Cm^R^*FLP*^+^λ*c*I857^+^	Datsenko and Wanner (2000)
pKD3	repR_R6K_Ap^R^ FRT Cm^R^ FRT	Datsenko and Wanner (2000)
pKD46	rep_pSC101_^ts^ Ap^R^ p*_araBAD_*γβ exo	Datsenko and Wanner (2000)
p*mgtP*-′*lacZ*	rep_p15A_ Cm^R^ p*_lac1-6_ mgtP*-′*lacZ*	This work
p*mgtP* stop-′*lacZ*	rep_p15A_ Cm^R^p*_lac1-6_ mgtP* stop-′*lacZ*	This work
pUHE21-2*lacI*^q^	rep_pMBI_ Ap^R^*lacI*^q^	Soncini *et al*. (1995)
p*mgtP*	pUHE21- *mgtP*	This work
p*supF*	pUHE21-s*upF*	Park *et al*. (2010)
pfpv25	pMB1ori, Ap^R^, promoterless *gfp*	Valdivia and Falkow (1996)
pGFP303	pfpv25 p*_mgtC_*-*mgtC* leader 303-*gfp*	Lee and Groisman (2012)
pGFP303 A_44-46_ → T	pfpv25 p*_mgtC_*-*mgtC* leader 303 (A_44-46_ → T)-*gfp*	Lee and Groisman (2012)
pGFP303 *mgtP*_pro→gly_	pfpv25 p*_mgtC_*-*mgtC* leader 303 (*mgtP*_pro→gly_)-*gfp*	This work
ptGFP	ColE1 ori Ap^R^*′gfp*	This work
ptGFP303	p*_mgtC_*-*mgtC* leader 303-*′gfp*	This work
ptGFP303 re211-214	p*_mgtC_*-*mgtC* leader 303 (re211-214)-*′gfp*	This work
ptGFP303 *mgtP* (CUG)	p*_mgtC_*-*mgtC* leader 303 (*mgtP*(CUG))-*′gfp*	This work
ptGFP303 *mgtP* (CUG) re211-214	p*_mgtC_*-*mgtC* leader 303 (*mgtP*(CUG) re211-214)-*′gfp*	This work

### Construction of plasmids harbouring a *lacZ* translational fusion to *mgtP*

Polymerase chain reaction (PCR) fragments corresponding to nucleotides 148–219 of the *mgtCBR* leader were amplified with primer 9804, which includes the sequence corresponding to the p*lac_1–6_* promoter, and either primer 9805 or 9806 (creating a stop codon) using 14028s genomic DNA as a template. The resulting PCR products were digested with SmaI and XbaI and cloned into plasmid pACYC-′*lacZ* digested with the same enzymes. The sequence of the resulting constructs was verified by DNA sequencing.

### Construction of a plasmid harbouring the *mgtP* ORF

Plasmid p*mgtP* was constructed as follows: a PCR fragment corresponding to the *mgtP* ORF generated by PCR with primers 8587 and 8589 using 14028s genomic DNA as a template, was digested with HindIII and BamHI and cloned into pUHE 21-2*lacI*^q^ digested with the same enzymes. The sequence of the resulting constructs was verified by DNA sequencing.

### Construction of plasmids harbouring fusions to a promoterless *gfp* gene

pGFP303, a *gfp* plasmid with the PhoP-dependent *mgtCBR* promoter and the wild-type *mgtC* leader, and its derivative, the A_44–46_→T substitutions in the *mgtC* leader were constructed as described (Lee and Groisman, 2012). Derivatives pGFP303 with nucleotide substitutions in the *mgtC* leader were constructed by cloning PCR fragments generated by two rounds of PCR reactions. For the *mgtP*_Pro→Gly_ substitution in the *mgtC* leader, a first PCR fragment was generated with primers 1746 and 8826, and a second fragment was generated with primers 8827 and 8117 and 14028s genomic DNA as a template. A third PCR was performed with primers 1746 and 8117 using the two PCR-generated DNA fragments as templates. The resulting PCR product was digested with EcoRI and XbaI and cloned into plasmid pfpv25 digested with the same enzymes. The sequence of the resulting construct was verified by DNA sequencing.

ptGFP, a *gfp* plasmid for translational fusion, was constructed as follows: a PCR fragment corresponding to the *gfp* gene starting from the third codon generated by PCR with primers 10109 and 10110 using pfpv25 plasmid as a template, was digested with BamHI and HindIII and cloned back into pfpv25 digested with the same enzymes, creating a *gfp* plasmid lacking its own RBS and the first two codons of the *gfp* gene (*′gfp*).

ptGFP303, a plasmid with the PhoP-dependent *mgtCBR* promoter, the wild-type *mgtCBR* leader and first two codons of *mgtC* fused in frame to the *gfp* gene was constructed as follows: a PCR fragment generated with primers 1746 and 10111 using 14028s genomic DNA as a template and digested with EcoRI and BamHI was cloned into plasmid ptGFP digested with the same enzymes.

Derivatives of ptGFP303 with nucleotide substitutions in the *mgtCBR* leader region were constructed by cloning PCR fragments generated by two rounds of PCR reactions. For the substitution in the position at 211–214 in the *mgtC* leader to hinder formation of stem-loop C, a first PCR fragment was generated with primers 1746 and 10113, and a second fragment was generated with primers 10112 and 10111 using 14028s genomic DNA as a template. A third PCR reaction was performed with primers 1746 and 10111 using the two PCR-generated DNA fragments as templates. The resulting PCR product was cloned into ptGFP using the same restriction enzymes used for construction of ptGFP303. All other substitutions were generated in a similar way using the following primer pairs: *mgtP* (CUG) (1746/8344 and 8347/8117) using 14028s genomic DNA as a template and *mgtP* (CUG) re211-214 (1746/10113 and 10112/10111) using ptGFP *mgtP* (CUG) plasmid as a template. DNA sequencing was used to verify the nucleotide sequences of all constructs.

### Construction of a strain with a chromosomal deletion of the *proB* gene

A *Salmonella* strain deleted for the *proB* gene was generated by the one-step gene inactivation method (Datsenko and Wanner, 2000). A Cm^R^ cassette was PCR amplified from plasmid pKD3 using primers 11729 and 11730 and the resulting PCR product was integrated into the 14028s chromosome to generate EL350 (*proB*::Cm^R^). A P22 phage lysate grown in strain EL350 was used to transduce EG9652 (*purB877*::Tn10) *Salmonella* selecting for chloramphenicol resistance to generate EL333 (*proB*::Cm^R^*purB877*::Tn*10*).

### Construction of strains with chromosomal mutations in the *mgtCBR* leader region

Two different methods were used to generate strains with chromosomal mutations in the *mgtCBR* leader. For strain EL379, we used the fusaric acid method as described (Lee and Groisman, 2010). DNA fragments carrying 10 out of 16 sense codons substitution in the *mgtP* were prepared by a two-step PCR reaction. For the first PCR reaction, we used two primer pairs 8118/11963 and 11962/7308, and 14028s genomic DNA as a template. For the second PCR reaction, we mixed two PCR products from the first PCR reaction as templates and amplified a DNA fragment using primers 8118 and 7308. The resulting PCR products were purified and integrated into the EG18715 chromosome and selected against Tet^R^ with media containing fusaric acid to generate EL379, a Tet^S^ Amp^S^ chromosomal mutant. The presence of the expected substitution was verified by sequencing.

To create mutations with the start codon or stop codons at different positions in *mgtP*, DNA fragments carrying the mutation at the start codon or stop codons in the *mgtP* were prepared as follows: we used primer pairs 8118/8344 and 8347/7308 (for CUG), 8118/8348 and 8349/7308 (for UAA2), 8118/8699 and 8698/7308 (for UAA3), 8118/8809 and 8808/7308 (for UAA4), 8118/8704 and 8703/7308 (for UGA5) or 8118/9853 and 9852/7308 (for UAG2) and 14028s genomic DNA as a template in the first PCR reaction. For the second PCR reaction, we mixed the two PCR products from the first PCR reaction as templates and amplified the DNA fragment with the expected substitutions using primers 8118 and 7308. The resulting PCR products were purified and integrated into the EG18798 chromosome and selected against Tet^R^ in media containing fusaric acid to generate strains EG18799, EG18801, EG19251, EG19272, EG19253 and EG19840, which were Tet^S^ Amp^S^.

All other chromosomal mutants with substitutions in the *mgtC* leader were constructed by a multiple step PCR process. Strain EL296 was constructed by inserting a Cm^R^ cassette in the *yicL* gene, which is 278 nt upstream from *mgtC* transcription start site. The Cm^R^ cassette was amplified from plasmid pKD3 using primers 4801 and 4802 and the resulting PCR products were integrated into the 14028s chromosome to generate EL296 (*yicL*::Cm^R^). Then, we prepared DNA fragments containing a Cm^R^ cassette and the proper nucleotide substitutions in the *mgtC* leader using two primer pairs and EL296 genomic DNA as a template: 10077/8826 and 8827/7308 for the *mgtP*_Pro10,11,12→Gly_ substitution; 10077/11732 and 11731/7308 for the *mgtP*_Pro12→Gly_ substitution; 10077/11734 and 11733/7308 for the *mgtP*_Pro10,11→Gly_ substitution; 10077/11736 and 11735/7308 for the *mgtP*_Pro10→Gly_ substitution; and 10077/11727 and 11726/7308 for the A_44–46_→T substitution in the *mgtCBR* leader. The two resulting DNA fragments from the first PCR reactions were mixed and used as PCR templates to amplify DNA fragments containing Cm^R^ cassette and the proper nucleotide substitution using primers 10077 and 7308. The resulting DNA fragments were purified and integrated into the 14028s chromosome by the one-step inactivation method (Datsenko and Wanner, 2000) and mutants were selected for resistance to chloramphenicol. The presence of the expected substitution was verified by DNA sequencing.

### Effect of exogenous adenine on gene expression

Experiment was carried out using the adenine auxotrophic strain EG9652 harbouring a plasmid harbouring the *mgtC*–*gfp* fusion (or the plasmid vector) as described (Lee and Groisman, 2012).

### Effect of proline limitation on gene expression

The proline limitation experiment was performed as described (Park *et al*., 2010) with the following modifications: proline auxotrophic strains with a wild-type or mutant *mgtC* leader were grown overnight in N-minimal medium containing 10 mM Mg^2+^, and 1 mM proline. 1/100 dilution of the overnight culture was used to inoculate 20 ml of the same medium and grown for 3 h. Cells were then washed and transferred to 20 ml of N-minimal medium containing 500 µM Mg^2+^ and 1 mM proline and grown for 1 h. The cells were harvested and washed with N-minimal medium containing 500 µM Mg^2+^ without proline and resuspended in a small volume of the same media. Then, the resuspended cells were split into two cultures in 10 ml of N-minimal medium containing a mixture of 19 amino acids (all essential amino acids except proline) and 500 µM Mg^2+^ with or without 1 mM proline and growth continued for 45 min. Bacteria were stabilized using RNAprotect Bacteria Reagent (Qiagen) and RNA was isolated for further analysis.

### Effect of leucine limitation on gene expression

Leucine limitation was performed as described above except that we used a leucine auxotroph and a 19 amino amino acid mixture (all essential amino acids except leucine).

### Effect of hyperosmotic stress on gene expression

Experiment was performed as described (Park *et al*., 2010).

### Effect of proline and/or adenine on gene expression

Proline and adenine auxotrophic strains were grown overnight in N-minimal medium containing 10 mM Mg^2+^, 1 mM proline and 250 µM adenine. 1/50 dilution of the overnight culture was used to inoculate 40 ml of the same medium and grown for 3 h. Cells were then washed and transferred to 40 ml of N-minimal medium containing 500 µM Mg^2+^, 1 mM proline and 25 µM adenine and grown for 1 h. The cells were harvested and washed with N-minimal medium containing 500 µM Mg^2+^ and 25 µM adenine without proline and resuspended in a small volume of the same media. Then, the resuspended cells were split into four cultures in 10 ml of N-minimal medium containing a mixture of 19 amino acids (all essential amino acids except proline) and 500 µM Mg^2+^ with or without 1 mM proline in the presence of 25 µM or 250 µM adenine and growth continued for 1 h. Bacteria were stabilized using RNAprotect Bacteria Reagent (Qiagen) and RNA was isolated for further analysis.

### Quantitative real-time PCR

Total RNA was isolated using RNeasy Kit (Qiagen) according to the manufacturer’s instructions. The purified RNA was quantified using a Nanodrop machine (NanoDrop Technologies). cDNA was synthesized using High Capacity RNA-to-cDNA Master Mix (Applied Biosystems). The mRNA levels of the *mgtC, mgtB, mgtA, phoP* and *rrs* genes were measured by quantification of cDNA using SYBR Green PCR Master Mix (Applied Biosystems, Foster City) and appropriate primers (*mgtC* leader: 6962/6963, *mgtC* coding: 7530/7531, *mgtB* coding: 7763/7764, *mgtA* leader: 7225/7226, *mgtA* coding: 4308/4309, and *phoP* coding: 4489/4490) and monitored using a Fast ABI7500 machine (Applied Biosystems, Foster City). Data were normalized to the levels of 16S ribosomal RNA amplified with primers 6970 and 6971.

### β-Galactosidase assays

Cells were grown overnight in N-minimal media and washed once in N-minimal media before resuspending them in N-minimal media with different MgCl_2_ concentrations for 4 h at 37°C with shaking. The activity was determined as described (Miller, 1972). Data correspond to two or more independent experiments conducted in duplicate.

### In-line probing

Experiments were carried out as described (Regulski and Breaker, 2008) with the following modifications: the *mgtC* leader RNA was synthesized *in vitro* with T7 RiboMAX Large Scale RNA production system (Promega) from the DNA template amplified from wild-type 14028s and primers 10336 and 6140 for the *mgtC* leader 196–385. To probe the structures at different Mg^2+^ concentrations, 1 pmol of 5′-end-labelled *mgtC* leader RNA was incubated in buffer [100 mM KCl, 50 mM Tris (pH 8.0)] with 1, 5 or 20 mM Mg^2+^ for 40 h at room temperature. Reactions were quenched with urea gel loading buffer II (Ambion) and analysed on a 10% denaturing polyacrylamide gel.
