# Supplementary material for: Tandem attenuators control expression of the Salmonella mgtCBR virulence operon
Source: Mol Microbiol. 2012 Oct;86(1):212–24. doi: 10.1111/j.1365-2958.2012.08188.x (PMC3641672; doi:10.1111/j.1365-2958.2012.08188.x)
Supplement: Supplementary file 1 [file mmi0086-0212-sd1.pdf]

Supporting Experimental procedures

Table S1. Primers Used in this Study

No.	Sequence (from 5′ to 3′)
1746	GGAATTCCTTTGCTCCATGATGTAC
4308	ACCGCGGTAAATGCGACTAT
4309	TGCCGCGACTTTCAGACA
4489	GATGAAGACGGCCTTTCCTTAA
4490	GAACCGGCAGTGAAACATCA
4801	AAAAGGGATGCTAAACGTCCTGATTGCCGCCGTATTGTGGATA TGAATATCCTCCTTAGT
4802	TTTGCTCCATGATGTACTGCGCGCAAACGCCTGAACTCCCGTG TAGGCTGGAGCTGCTTC
6140	TACGTTGACGCCATTGTCTTTC
6962	GCAGGAGTAATATGTTGGACAGTCAC
6963	GGGAGATTGCTGCCCACC
6970	CCAGCAGCCGCGGTAAT
6971	TTTACGCCCAGTAATTCCGATT
7225	TTCAGGGTCCATGTCGCC
7226	CCACAAAACTTATGGATTTATGCGT
7308	ATTGGCGCAAAGAATAATGATCG
7530	CAGCCCGCGCACATTC
7531	TTGTCTCTGGGATTGGCTTTCT
7763	TCAGAAAATGATAAGCAGCATAAAAAA
7764	CCCTGACGATGGCTGTTCA

8117	AAGTCTAGATTAACATACGTTCCTCCATT
8118	TACGTGCAGGCATCATAACAGAGC
8344	AGCGTGTTTAAACAGGAACAGGACGATTACCTCGC
8347	GCGAGGTAATCGTCCTGTTCCTGTTTAAACACGCT
8348	GGAGGAAATAAAGCTTATTTAAACATGAACA
8349	TGTTCATGTTTAAATAAGCTTTATTTCCTCC
8587	GATCCATGTTCATGTTTAAACACGCTTTATTTCCTCCGCCGTTAACACGACGCTAAA
8589	AGCTTTTAGCGTCGTGTTAACGGCGGAGGAAATAAAGCGTGTTTAAACATGAACATG
8698	ATGTTTAAACACGCTTTATTTAACCGCCGTTAACACGACGCTA
8699	TAGCGTCGTGTTAACGGCGGTTAAAATAAAGCGTGTTTAAACAT
8703	CCTCCGCCGTTAACATGACGCTAATTGCCTCAG
8704	CTGAGGCAATTAGCGTCATGTTAACGGCGGAGG
8808	CACGCTTTATTTCCTCCGCCGTAAACACGACGCTAATTGCCTCAG
8809	CTGAGGCAATTAGCGTCGTGTTTACGGCGGAGGAAATAAAGCGTG
8827	GTTTAAACACGCTTTATTTGGCGGCGGCTTAACACGACGCTAATTGC
9804	GCTCTAGACTTTACACTTTAAGCTTTTTATGTTTATGTTGTGTGGAGCGCATTGTCGCGAGGTAATCG

9805	GTCCCGGGGCGTCGTGTTAACGGCGGAGGAAATAAAGCGTGT TTAAACAT
9806	GTCCCGGGGCGTCGTGTTAACGGCGGAGGAAATAAAGCCTAT TTAAACAT
9852	ATGTTCATGTTTAAATAGGCTTTATTTCCTCCG
9853	CGGAGGAAATAAAGCCTATTTAAACATGAACAT
10077	ACGTATCATC GTCAGGAACTG
10109	CGCGGATCCAGTAAAGGAGAAGAACTTTTCACTGG
10110	ATTAAGCTTGCATGCCTGCAGGAGATTTAT
10111	CGCGGATCCTAACATACGTTCCTCCATTT
10112	CCTCCGCCGTTATGTGGACGCTAATTGC
10113	GCAATTAGCGTCCACATAACGGCGGAGG
10336	TAATACGACTCACTATAGGG TTTCCTCCGCCGTTAACACG
11726	GGACAGTCACTTTTACGTTTTTCATCTGGCAAGTT
11727	AACTTGCCAGATGAAAAACGTAAAAGTGACTGTCC
11729	TCATTTGGTCTTTATTTCTTATTTTGAACGGCAGAGAATCGTGT AGGCTGGAGCTGCTTC
11730	TTAGCAGCAATGCCCATTTGTTCCAGCATATGCCTGTTCCATAT GAATATCCTCCTTAG
11731	GTTTAAACACGCTTTATTTCCTCCGGGCTTAACACGACGCTAA TTGC
11732	GCAATTAGCGTCGTGTTAAGCCCGGAGGAAATAAAGCGTGTTT AAAC
11733	GTTTAAACACGCTTTATTTGGCGGCCCGTTAACACGACGCTAA

	TTGC
11734	GCAATTAGCGTCGTGTTAACGGGCCGCCAAATAAAGCGTGTTTAAAC
11735	GTTTAAACACGCTTTATTTGGCCCGCCGTTAACACGACGCTAATTGC
11736	GCAATTAGCGTCGTGTTAACGGCGGGCCAAATAAAGCGTGTTTAAAC
11962	CATTGTCGCGAGGTAATCGTCATGCTGCTGTATCTTTTCACTTTCTTACCTCCAGGTATGACACGACGCTAATTGCCTCAG
11963	CTGAGGCAATTAGCGTCGTGTCATACCTGGAGGTAAGAAAGTGAAAAGATACAGCAGCATGACGATTACCTCGCGACAATG

Supplemental figure legends

Fig. S1. The possibility of forming the alternative stem-loops C and D versus stem-loop E is conserved in the *mgtCBR* leader from *S. enterica*, *S. marcescens*, and *P. asymbiotica*. The predicted ribosome-binding site (RBS) and *mgtC* start codon are boxed.

Fig. S2. Stem-loops C and D can form *in vitro*. In-line probing analysis of 5′ ^{32}P-labeled RNA corresponding to nucleotides 196-385 from the wild-type *mgtCBR* leader. NR, T1, and OH identify rows containing untreated RNA, or RNA subjected to partial digestion with the ribonuclease T1 (which cleaves at unpaired G-residues) or alkali (OH), respectively. Remaining lanes correspond to RNA incubated in the presence of 1, 5 and 20 mM Mg^{2+} as described in *Experimental procedures*. Lines with numbers correspond to the regions shown at the bottom of the figure.

Fig. S3. The *mgtP* ORF is translated *in vivo*. β-galactosidase activity (Miller units) produced by wild-type *Salmonella* (14028s) harboring either the plasmid vector (pACYC-'*lac*Z), or derivatives with a *lac* translational fusion to the last *mgtP* sense codon (p*mgtP*-'*lac*Z), or following the last (i.e., stop) *mgtP* codon (p*mgtP*(uag)-'*lac*Z). Bacteria were grown in N-minimal media with 10 μM (L) or 10 mM (H) Mg^{2+} for 4 h as described in *Experimental procedures*.

Lee_Fig. S1

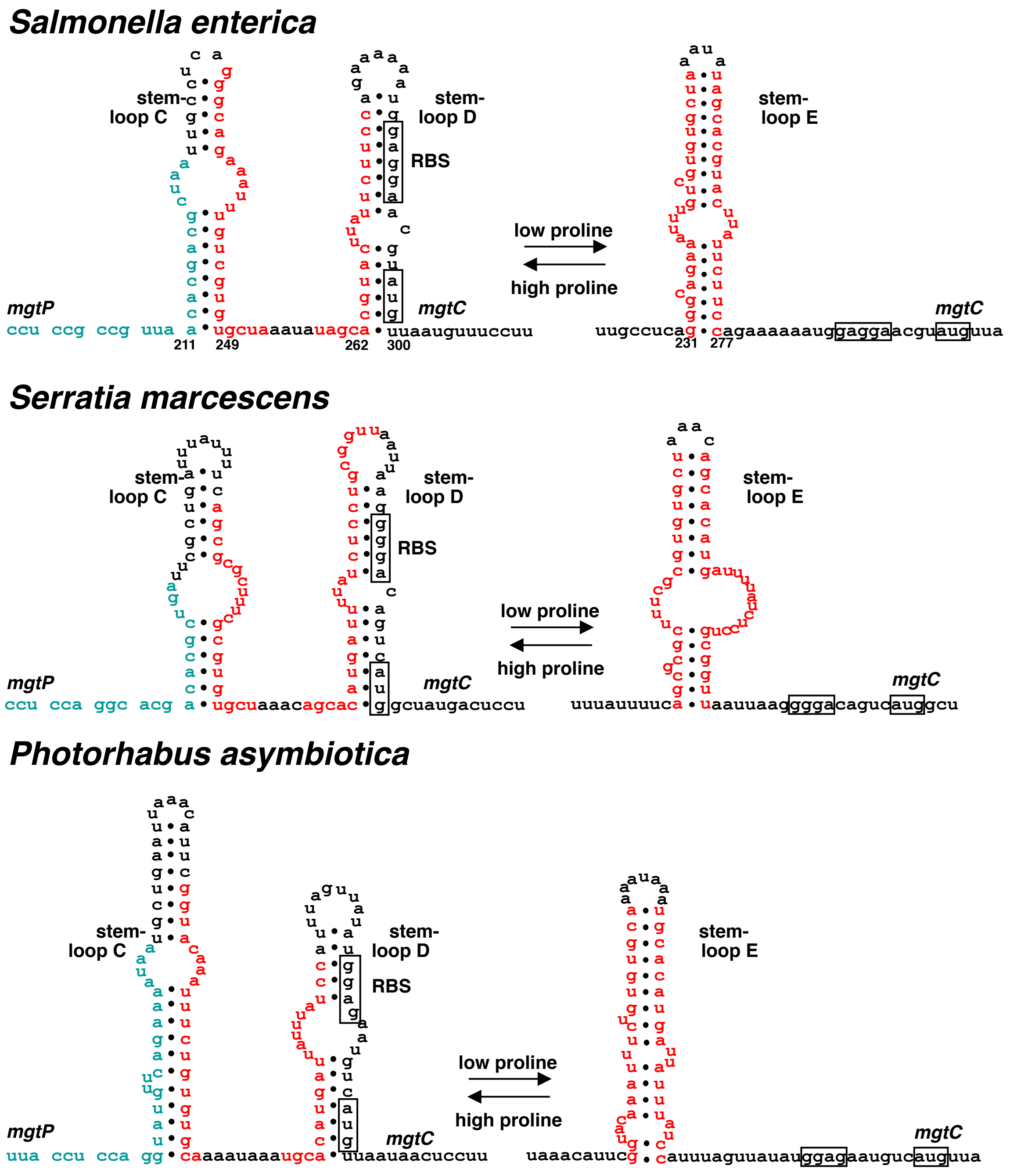

Salmonella enterica
stem-loop C
stem-loop D
RBS
stem-loop E
low proline
high proline
mgtP
mgtC
211
249
262
300
231
277
Serratia marcescens
stem-loop C
stem-loop D
RBS
stem-loop E
low proline
high proline
mgtP
mgtC
Photorhabus asymbiotica
stem-loop C
stem-loop D
RBS
stem-loop E
low proline
high proline
mgtP
mgtC

Lee_Fig. S2

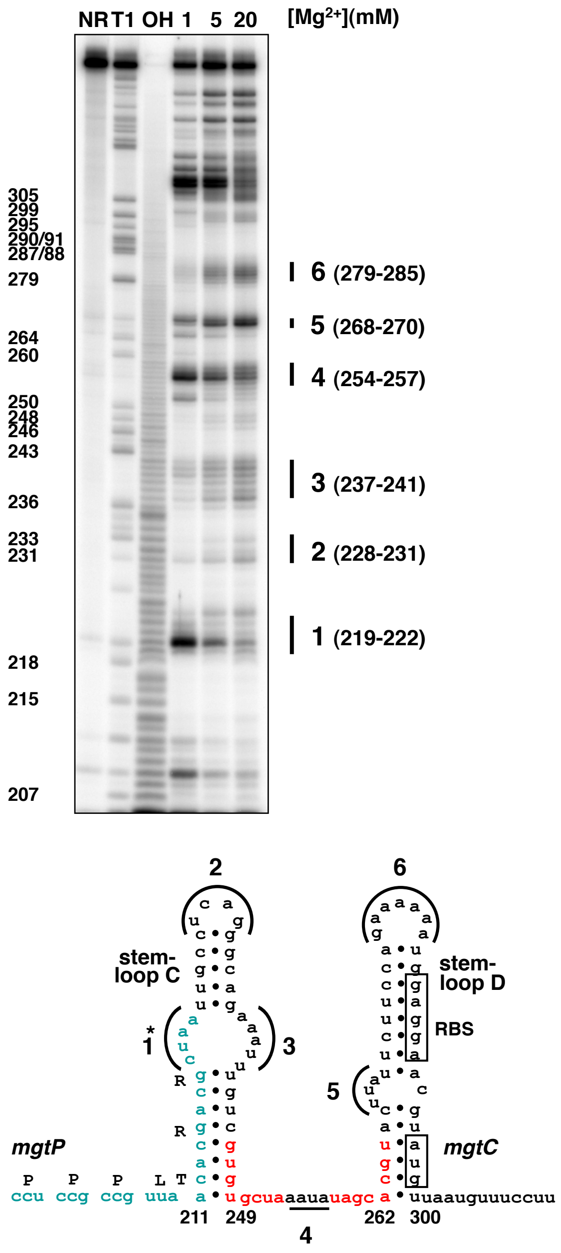

NR T1 OH 1 5 20 [Mg2+](mM)
305
299
295
290/91
287/88
279
264
260
250
248
246
243
236
233
231
218
215
207
6 (279-285)
5 (268-270)
4 (254-257)
3 (237-241)
2 (228-231)
1 (219-222)
stem-loop C
stem-loop D
RBS
mgtP
mgtC
P P P L T
ccu ccg ccg uua
uuaauguuuccuu
211
249
262
300

Lee_Fig. S3

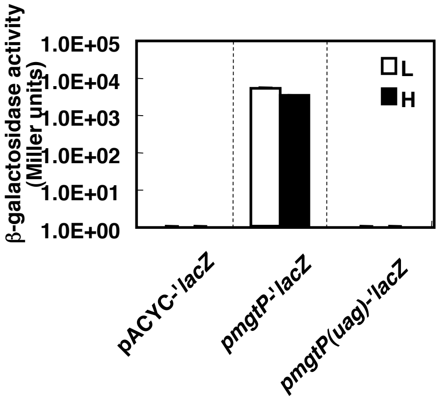
β-galactosidase activity
(Miller units)
1.0E+05
1.0E+04
1.0E+03
1.0E+02
1.0E+01
1.0E+00
L
H
pACYC-'lacZ
pmgtP-'lacZ
pmgtP(uag)-'lacZ
